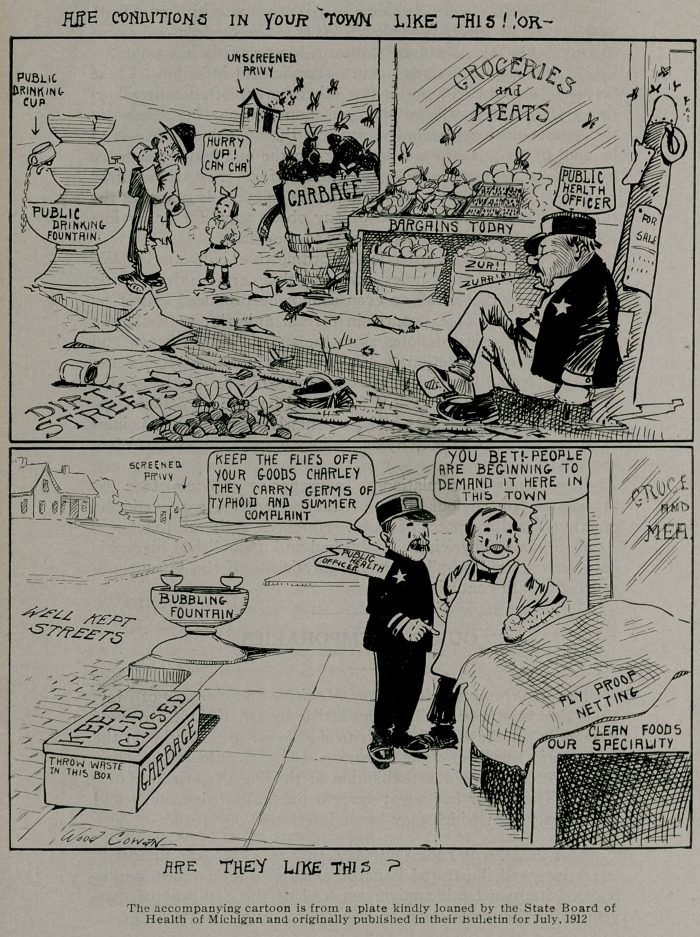# Our Contemporaries

**Published:** 1912-09

**Authors:** 


### OUR CONTEMPORARIES.

---

The *Old Dominion Journal of Medicine and Surgery* of May, 1912, comments editorially on an article by Dr. F. D. Snyder, of Ashtabula, in the *Cleveland Medical Journal,* of March, on the Prehistoric Surgeon. The Peruvian skulls, mentioned, showing trephine marks, were on exhibition at the Pan-American Exposition. The editor has a government publication describing and illustrating them which may be of interest to our readers.

---

*N. A. R. D. Notes* for Thursday, July 25, 1912, is an unusually attractive, well illustrated issue of extra size in commemoration both of its own tenth anniversary and of the 14th annual convention of the Association at Milwaukee, Aug. 12, 1912.

The accompanying cartoon is from a plate kindly loaned by the State Board of Health of Michigan and originally published in their Bulletin for July, 1912

The *Homoeopathic Envoy*, of August, reproduces, under the title of "The Allopathic Point of View," our rejoinder to "Jane" of the *Buffalo Express* (see January issue) in which we insisted on the necessity of certain harsh methods like vaccination. The *Envoy* says:

But, kind Sir, if there are "nice, easy ways" of getting people well, why should not the people have the benefit? Homoeopathy is a nice, easy way and the statistics of a hundred years show that it is more efficacious than your harsher way, yet you would have none of it did not the public intervene. Learn wisdom, brother!

In reply, we would like to have it distinctly understood that while we have many homeopathic friends and have always had a high regard for Hahnemann—with due allowances for the times in which he lived—we do not know any allopaths and would have a club for them if there were any. This is not merely a quibble of words. If there were a medical sect upholding the notion of giving big doses, of giving drugs whose symptoms should be directly opposite to those of the patient, we should oppose that sect much more than we ever have homoeopaths, for such a sect would do damage in a very direct manner. Our original article had no reference to homoeopathy at all. We as editor or practitioner, are always looking for "nice easy ways" of getting people well and think we know quite a number but we regard the "getting well" as more important than the "nice and easy."

The writer has been vaccinated himself once successfully and two or three times unsuccessfully, to insure protection. He has vaccinated several thousand others and has never seen but one case in which it could not be regarded as "nice and easy." That case was a dirty little girl at school and she had a sore arm but nothing alarming. Why the *Envoy* should object to vaccination, we can not understand. It conforms in everything but detail to Hahnemann's conception of the itchmite as a factor of disease. This conception, as an eighteenth century precursory hypothesis of the micro-organismic origin of disease, is a wonderful indication of Hahnemann's scientific imagination and logic. As a literal dictum it is absolutely contrary to demonstrated fact. Vaccination is in conformity with the general conception of "proving" a remedial agent. It is strictly though broadly in accordance with the idea that "similia similibus curantur." The methods of producing vaccine correspond closely to the ideas of developing potency by successive inoculations and dilutions and to that of "grafts." It is quite in line with the use of tears, pus, discharges, etc., listed by homoeopathic firms within recent years, if not up to the present. And, best of all, vaccination has proved its success to the great majority of expert investigators and students of statistics.

---

The *British and Colonial Druggist* of June 7, 1912, continues the "Post Graduate" lectures of the Pharmaceutical Society of Great Britain. This course of lectures is of high scientific value and the gratitude of both the British and other pharmacists and physicians is due to Mr. S. W. Fairchild of New York City, for donating the funds necessary to secure it. The first lecture was given by Sir A. W. Tilden, the next two by Prof. W. H. Perkin and the fourth and fifth by John C. Umney, the President-elect of the British Pharmaceutic Conference.

## Figures and Tables

**Figure f1:**